# Inter-domain electron transfer in cellobiose dehydrogenase: modulation by pH and divalent cations

**DOI:** 10.1111/febs.13310

**Published:** 2015-05-16

**Authors:** Daniel Kracher, Kawah Zahma, Christopher Schulz, Christoph Sygmund, Lo Gorton, Roland Ludwig

**Affiliations:** 1 Department of Food Sciences and Technology, Food Biotechnology Laboratory, University of Natural Resources and Life SciencesVienna, Austria; 2 Department of Analytical Chemistry, Biochemistry and Structural Biology, Lund UniversitySweden

**Keywords:** cellobiose dehydrogenase, divalent cation bridging effect, domain docking, inter-domain electron transfer, oxidative cellulose degradation

## Abstract

The flavocytochrome cellobiose dehydrogenase (CDH) is secreted by wood-decomposing fungi, and is the only known extracellular enzyme with the characteristics of an electron transfer protein. Its proposed function is reduction of lytic polysaccharide mono-oxygenase for subsequent cellulose depolymerization. Electrons are transferred from FADH_2_ in the catalytic flavodehydrogenase domain of CDH to haem *b* in a mobile cytochrome domain, which acts as a mediator and transfers electrons towards the active site of lytic polysaccharide mono-oxygenase to activate oxygen. This vital role of the cytochrome domain is little understood, e.g. why do CDHs exhibit different pH optima and rates for inter-domain electron transfer (IET)? This study uses kinetic techniques and docking to assess the interaction of both domains and the resulting IET with regard to pH and ions. The results show that the reported elimination of IET at neutral or alkaline pH is caused by electrostatic repulsion, which prevents adoption of the closed conformation of CDH. Divalent alkali earth metal cations are shown to exert a bridging effect between the domains at concentrations of > 3 mm, thereby neutralizing electrostatic repulsion and increasing IET rates. The necessary high ion concentration, together with the docking results, show that this effect is not caused by specific cation binding sites, but by various clusters of Asp, Glu, Asn, Gln and the haem *b* propionate group at the domain interface. The results show that a closed conformation of both CDH domains is necessary for IET, but the closed conformation also increases the FAD reduction rate by an electron pulling effect.

## Introduction

Cellobiose dehydrogenase (CDH; EC 1.1.99.18; CAZy database ID AA3-1) is detected in the secretome of wood-degrading, composting and plant pathogenic fungi. The results of recent studies support a role in the oxidative breakdown of crystalline cellulose or other polymeric, carbohydrate constituents of plant biomass in combination with lytic polysaccharide monooxygenase (CAZy database ID AA9) 1–5. As a flavocytochrome, CDH exhibits a unique architecture for a secreted enzyme. It features an N-terminal electron-transferring cytochrome domain (CYT) that is bound to a carbohydrate-oxidizing dehydrogenase domain (DH). Electrons obtained from carbohydrate oxidation are transferred from the DH to the CYT by inter-domain electron transfer (IET). The proposed function of the CYT is to donate electrons to the copper centre of lytic polysaccharide mono-oxygenase to activate oxygen 2,6. IET from FADH_2_ to the *b*-type haem in the CYT is therefore an important step towards oxidative cellulose depolymerization.

Various pH optima for the IET have been reported in the literature. Usually, the activity with the *in vitro* electron acceptor cytochrome *c* (cyt *c*), which only interacts with the CYT 6–8, is used to approximately determine the IET. Direct observation of IET requires stopped-flow spectrophotometry, which has been performed for *Phanerochaete chrysosporium* CDH 9. These studies show that CDHs from basidiomycetes (class I) have acidic pH optima, and IET ceases at approximately pH 6. The reported reason is electrostatic repulsion between the domains 10. Ascomycetous CDHs (class II) are more diverse, and some of them exhibit less acidic or even alkaline IET pH optima 10,11. This is remarkable because the reported isoelectric points for these CDHs do not differ from those of basidiomycetous CDHs with acidic pH optima. A possible explanation is that the DH and the CYT exhibit different patches of charged or non-charged amino acid residues.

The structure of full-length CDH is unknown, but structures for the isolated DH (PDB ID 1KDG) and CYT (PDB ID 1D7C) from *P. chrysosporium* are available 12,13. Docking of the two domains has been reported, whereby a haem propionyl group was found to protrude into the substrate channel of the DH and come into close contact with the FAD 10. The modelling is based on structural complementarity, and it is unclear whether this closed conformation is energetically favoured or whether the CYT is mobile and moves freely between a closed and an open conformation. The long linker (15 amino acids) between the domains suggests that the CYT has a high degree of freedom to move when not in contact with the DH. This appears to be necessary on order to transfer electrons to proteins such as cyt *c* or lytic polysaccharide mono-oxygenase. In previous stopped-flow measurements 9, no IET above pH 6 was observed for *P. chrysosporium* CDH, but the dehydrogenase domain was highly active at alkaline pH. One of two explanations may apply: (i) the domains separate because of electrostatic repulsion, and the increased distance between the domains prevents IET, or (ii) the closed conformation is stable and the domains are kept together, but IET is disrupted by structural and protonation changes along the electron transfer pathway. The possibility of reversal of the electron transfer by shifting the FAD redox potential to above the haem *b* potential has been excluded by Igarashi *et al*. 9.

A recent study revealed a way to investigate the pH dependency of the IET in more detail. It reported that calcium ions increased the enzymatic activity and catalytic current of three CDHs from *Myriococcum thermophilum*, *Humicola insolens* and *Phanerochaete sordida* 14. Addition of a millimolar concentration of calcium ions to the buffer solution increased the catalytic current of CDH-modified spectroscopic graphite electrodes and also the turnover of cyt *c*. It was speculated that calcium ions modulate the domain interaction, and thereby enhance the IET between FAD and haem *b* cofactors. Here, we study the effect of the calcium concentration on twelve CDHs from various fungi to determine whether the effect is ubiquitous among CDH. We also investigate whether CDH is only IET-competent when both domains are in the closed conformation, and whether electrostatic repulsion above a certain pH prevents the closed state and therefore IET. *M. thermophilum* CDH was used to investigate the domain interaction and IET using spectroscopic, steady-state, pre-steady-state and calorimetric techniques. To elucidate the charges at the domain interfaces of three CDHs, comparative modelling and docking were performed.

## Results and Discussion

### Modulation of CDH activity by pH ions

**Figure 1:**
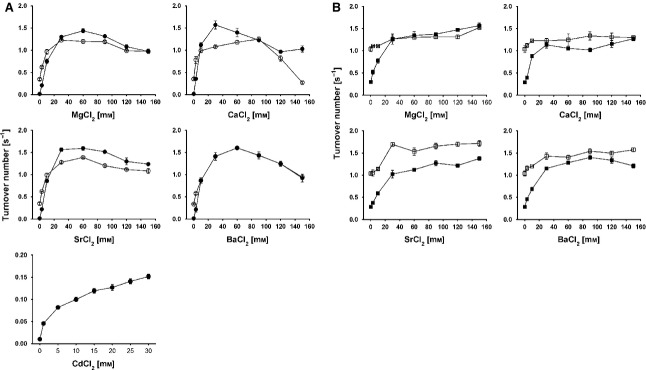
(A) Effect of divalent cation concentration on cyt *c* turnover for *Mt*CDH measured at pH 5.5 (open circles) and pH 7.5 (closed circles). (B) Effect of divalent cation concentration on turnover of the two-electron acceptor DCIP for *Mt*CDH at pH 5.5 (open squares) and pH 7.5 (closed squares).

CDH from *M. thermophilum* (*Mt*CDH), was previously reported to be responsive to calcium ions 14. A millimolar concentration of calcium ions increased the activity of the enzyme fivefold. Using a screening approach based on artificial electron acceptors for *Mt*CDH, we also observed such enhancement when using other divalent earth alkaline metals such as Mg^2+^, Sr^2+^, Ba^2+^ and Cd^2+^ (Fig.1), but not when using monovalent cations or anionic species (Table S1). The effect is stronger at alkaline pH, and is only observed for CDH activity towards cyt *c*, and to a much lesser extent for reduction of the two-electron acceptor 2,6-dichloroindophenol (DCIP). Reduction of the two-electron acceptor 1,4-benzoquinone (BQ) was not affected at all. Cyt *c* is solely reduced by the haem *b* of CDH and requires IET. The two-electron acceptors DCIP and BQ are reduced directly by FADH_2_ and do not require IET and the cytochrome domain. The increase in electron acceptor turnover in the presence of divalent cations depends on their concentration. Below a 3 mm concentration, only a small increase in cyt *c* turnover by CDH was detected. The maximal effect was found at concentrations between 30 and 60 mm, while higher concentrations also decreased the turnover of cyt *c* (Fig.1A). A smaller increase was found for DCIP turnover, where the optimum cation concentration was also 30–60 mm, but no decrease at higher concentrations was observed (Fig.1B). Apparent catalytic constants of *Mt*CDH for cyt *c* in the presence of 30 mm of divalent cations were measured at pH 5.5 and 7.5, and compared to a 30 mm concentration of Na^+^ (Table1). The increase in *k*_cat_ caused by divalent cations was 3.5–4-fold at pH 5.5, but 160–200-fold at pH 7.5. The huge enhancement at pH 7.5 is only partly attributable to an increase in the absolute turnover of cyt *c* by CDH (1.2–1.4-fold higher compared to pH 5.5), and is mostly due to a reduced of cyt *c* turnover in the absence of Ca^2+^. At pH 7.5 and in the absence of divalent cations (Na^+^ was used as substitute), cyt *c* turnover is negligible. In the presence of divalent cations, the catalytic efficiency for cyt *c* is very similar at pH 5.5 (mean of ∼ 2.8 × 10^5^ m^−1^·s^−1^) and pH 7.5 (mean of ∼ 3.2 × 10^5^ m^−1^·s^−1^). This indicates that divalent cations strongly counteract the shutdown of the IET at neutral/alkaline pH, and do not influence electron transfer from the CYT to cyt *c*. Apparently, neither the atomic radius (Ba^2+^ > Sr^2+^ > Ca^2+^ > Mg^2+^) nor the electronegativity (Mg^2+^ > Ca^2+^ > Sr^2+^ > Ba^2+^) of the earth alkali metal ions significantly modulate the observed enhancing effect. A lower enhancing effect was found for Cd^2+^, which increases cyt *c* turnover at pH 7.5 to 0.15 s^−1^, compared with a turnover of 1.3 s^−1^ observed in the presence of Ca^2+^ (Fig.1A).

**Table 1:** Catalytic constants of *Mt*CDH for cyt *c* measured in the presence of cations at a concentration of 30 mm

Salt/pH	*K*_M_ (μm)	*k*_cat_ (s^−1^)	*k*_cat_/*K*_M_ (m^−1^·s^−1^)
NaCl, pH 5.5	2.8 ± 0.5	0.4 ± 0.1	1.43 × 10^5^
NaCl, pH 7.5	0.5 ± 0.1	0.010 ± 0.003	0.20 × 10^5^
MgCl_2_, pH 5.5	5.6 ± 0.2	1.6 ± 0.1	2.86 × 10^5^
MgCl_2_, pH 7.5	6.1 ± 0.1	2.1 ± 0.1	3.44 × 10^5^
CaCl_2_, pH 5.5	5.6 ± 0.1	1.4 ± 0.1	2.50 × 10^5^
CaCl_2_, pH 7.5	6.0 ± 0.4	1.6 ± 0.2	2.67 × 10^5^
SrCl_2_, pH 5.5	4.3 ± 0.1	1.4 ± 0.4	3.26 × 10^5^
SrCl_2_, pH 7.5	5.8 ± 0.1	2.0 ± 0.1	3.45 × 10^5^
BaCl_2_, pH 5.5	5.5 ± 0.2	1.5 ± 0.1	2.73 × 10^5^
BaCl_2_, pH 7.5	6.5 ± 0.2	2.0 ± 0.1	3.08 × 10^5^

### Modulation of activity in CDH from other sources

Twelve CDHs from 11 fungi were subjected to screening using the three electron acceptors cyt *c*, DCIP and BQ at three pH values in the presence or absence of 30 mm Ca^2+^ (Tables2 and 3). None of the investigated enzymes showed activation of BQ turnover in the presence of Ca^2+^. DCIP turnover was slightly increased for some class II CDHs at pH 7.5. However, the turnover of cyt *c* was increased for both CDH classes, typically 1.4-fold for class I and between 1.3- and 4.2-fold for class II. At higher pH, the observed increase was most often higher, ∼ 2–4-fold for class I CDHs and 3–5-fold for class II CDHs. In contrast to ascomycetous class II CDHs, which show higher pH optima, the measured pH for class I CDHs was pH 6.5, and no activity for cyt *c* was detected at pH 7.5. Interestingly, there are exceptions from the observed increase in activity: The class I CDH from *Sclerotium rolfsii* showed a decreased cyt *c* turnover at pH 6.5, and the class II *Neurospora crassa* CDH IIA showed a decreased cyt *c* turnover at pH 5.5 and 7.5 in the presence of Ca^2+^. Whether these enzymes are inert to the presence of divalent cations, or the pH chosen was not suitable, cannot currently be determined. The 125-fold activation of *Mt*CDH activity at pH 7.5 in the presence of Ca^2+^ is very high compared to the other investigated CDHs. Despite these exceptions, the enhancement effect of divalent metal cations was observed for all tested CDH enzymes.

**Table 2:** Effect of 30 mm CaCl_2_ on CDHs from Ascomycota

		Activation (fold)								
		DCIP		BQ		Cyt *c*		pH optimum		
	Source	pH 5.5	pH 7.5	pH 5.5	pH 7.5	pH 5.5	pH 7.5	DCIP	Cyt *c*	pI
*Myriococcum thermophilum* IIA CBS 208.89	Recombinant	1.2	3.8	1.1	1.1	4.2	125	5.5	5.0	3.8
*Neurospora crassa* IIA CBS 232.56	Recombinant	1.1	1.1	1.0	0.9	0.5	0.7	5.0	6.0	4.9
*Neurospora crassa* IIB CBS 232.56	Recombinant	1.0	1.8	1.0	1.0	1.3	4.5	5.0	5.5	5.1
*Corynascus thermophilus* IIB CBS 174.70	Wild-type	1.4	4.0	1.0	1.1	2.6	3.0	5.0	7.5	3.8
*Chaetomium atrobrunneum* IIA CBS 238.71	Wild-type	1.1	3.2	0.9	0.9	1.0	5.2	6.0	5.0	4.1
*Hypoxolon haematostroma* IIB CBS 255.63	Wild-type	0.9	0.8	1.1	1.1	0.8	3.2	5.0	5.5	4.3
*Dichomera saubinetii* IIA CBS 990.7	Wild-type	1.0	0.9	1.0	1.2	1.4	4.7	5.5	5.5	4.2

**Table 3:** Effect of 30 mm CaCl_2_ on CDHs from Basidiomycota

		‘Activation’ (fold)								
		DCIP		BQ		Cyt *c*		pH optimum		
	Source	pH 4.5	pH 6.5	pH 4.5	pH 6.5	pH 4.5	pH 6.5	DCIP	cyt *c*	pI
*Trametes villosa* CBS 334.49	Wild-type	0.9	0.8	1.0	0.8	1.0	2.9	5.0	3.5	4.4
*Phanerochaete chrysosporium* K3	Wild-type	0.8	1.0	1.0	0.9	1.4	2.9	4.0	4.0	4.2
*Phanerochaete sordida* MB 66	Wild-type	0.9	0.9	0.9	0.9	1.4	2.2	4.0	4.0	4.1
*Sclerotium rolfisii* CBS 191.62	Wild-type	0.9	0.9	1.0	0.9	1.5	0.7	4.0	3.5	4.2
*Ceriporiopsis subvermispora* FP-90031	Wild-type	0.9	0.9	1.0	0.9	1.4	3.8	4.5	3.5	3.0

Values are means from three replicates; errors are below 10%.

### Modulation of electron acceptor pH profiles by Ca^2+^

**Figure 2:**
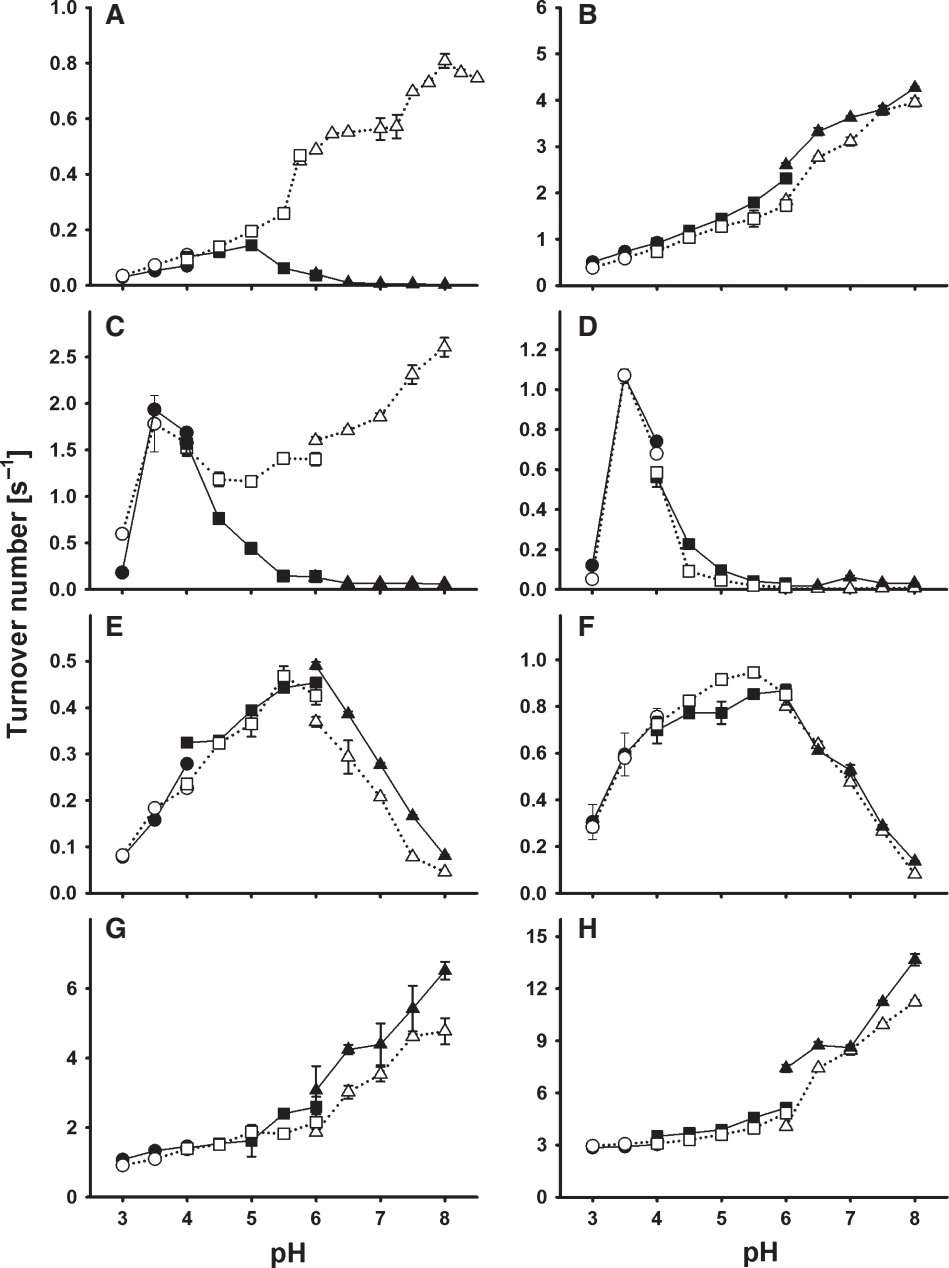
pH-dependent activities of *Mt*CDH (A–C, E, G) and the dehydrogenase domain *Mt*DH (D, F, H) towards the electron acceptors cyt *c* (A), ferrocenium (B), ferricyanide (C, D), DCIP (E, F) and 1,4-benzoquinone (G, H) measured in the absence of CaCl_2_ (open symbols) and upon addition of 30 mm CaCl_2_ (closed symbols). The buffers used were 50 mm sodium formiate (pH 3–4, circles), 50 mm sodium acetate (pH 4–6, squares) and 50 mm MOPS (pH 6–8, triangles). Error bars represent the standard deviation from three independent repeats.

The observed pH dependency of the electron acceptor turnover in the presence of divalent cations was studied in detail using *Mt*CDH and Ca^2+^ *partes pro toto* (Fig.2). The turnover rate of the positively charged one-electron acceptor cyt *c* by the CYT depends on the IET, and is optimum at pH 5. Above this pH, the turnover of cyt *c* decreases rapidly, and disappears at pH 6.5. As the charge of cyt *c* (pI 10.0–10.4) is not altered at this pH, the change in turnover is probably attributable to reduction of the IET between the DH and the CYT. At pH 5, the side-chain carboxy functions of surface-exposed aspartic acid (p*K*_a_ = 3.86) and glutamic acid (p*K*_a_ = 4.07) are ∼ 90% deprotonated, creating a strong electrostatic repulsion between both domains. In the presence of Ca^2+^, this reduction of turnover above pH 5 is not observed, indicating that the electrostatic repulsion is neutralized. The increasing turnover of cyt *c* at increasing pH shows that the reductive cycle at the FAD has an alkaline pH optimum.

The positively charged electron acceptor ferrocenium showed similar behaviour in the absence or presence of Ca^2+^ Increasing turnover at alkaline pH indicates that this one-electron acceptor does not depend on IET, but is reduced by the FADH_2_ directly. The activity of CDH towards the two-electron acceptors DCIP (with a strong negative partial charge) and BQ (with weak negative partial charge) was slightly increased in the presence of Ca^2+^ at pH values above 6. Interestingly, this activation was not observed for the single DH, which indicates that not only do the negative charges around the substrate channel reduce the accessibility for electron acceptors, but that the proximity of the haem *b* also affects electron acceptor-mediated oxidation of FAD.

The most interesting behaviour was observed for the ferricyanide anion. This one-electron acceptor may be directly reduced by FADH_2_ in the DH, but only at pH values below 5.0. Such behaviour was also observed for glucose oxidase 15. For CDH, ferricyanide reduction may be observed up to pH 6.0, demonstrating that the proximity of the CYT to the DH influences ferricyanide reduction more strongly than it influences DCIP or BQ reduction. The pH profiles of the DH in the presence and absence of Ca^2+^ reveal that the charge-induced reduced accessibility of the ferricyanide anion to the active site is a minor factor. The increasing ferricyanide turnover by CDH at alkaline pH in the presence of Ca^2+^ may be explained by two models. In model 1, the close proximity of the haem *b* to the FAD increases ferricyanide turnover at FAD. In model 2, the reduction of ferricyanide occurs at the CYT above pH 5. The two pH optima observed in the pH profile promote the second mechanism.

The breakdown of ferricyanide and cyt *c* reduction above pH 6.0–6.5 provides a measure for shutdown of the IET. If the pH increases above pH 6.5, the CYT of *Mt*CDH no longer interacts with the DH, because electrostatic repulsion of the negatively charged domains prevents adoption of a closed-state conformation (Table2, pI values). In the presence of Ca^2+^, the ferricyanide turnover of CDH shows a monotonic increase from pH 5–8. The pH profiles of ferricyanide and cyt *c* therefore give a clear indication that divalent cations support adoption of a closed conformation by the DH and the CYT when the domain interface becomes negatively charged at neutral/alkaline pH. This effect is not caused by the presence of distinct binding sites for divalent cations, as these were not found in our docking models of the DH and the CYT, and the 30–60 mm concentration of dications required to achieve the optimal effect is magnitudes higher than the reported affinities for Ca^2+^specific EF-hand binding sites, for example (2.1 μm 16 or 0.49 μm 17). The high concentration required suggests unspecific shielding of close opposing charges at both sides of the domain interface. Similar behaviour has been reported for surfactants, DNA and hyaluronan in the presence of Ca^2+^, and was termed the ‘divalent cation bridging effect’ 18–22.

### Spectral features of *Mt*CDH

**Figure 3:**
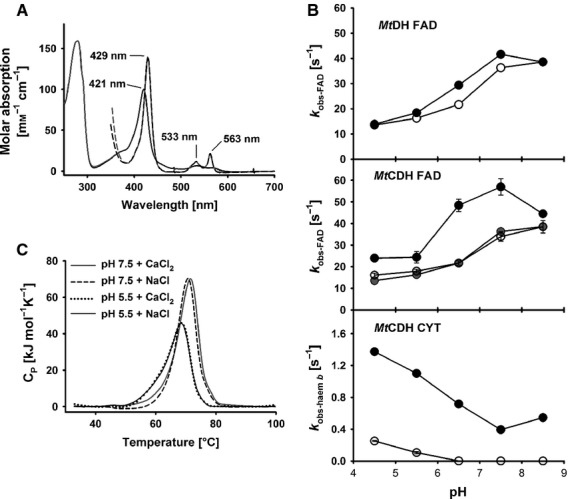
(A) Spectra of oxidized *Mt*CDH at pH 7.5 (50 mm MOPS) in the presence of 30 mm NaCl (black line) and 30 mm CaCl_2_ (grey line). Spectra of reduced MtCDH in the presence of NaCl (black dashed line) and CaCl_2_ (grey dashed line) were obtained upon addition of a 2000-fold excess of sodium dithionite. Under these conditions, the experimentally determined molar absorption coefficient of oxidized *Mt*CDH at 421 nm is 99.4 mm^−1^ cm^−1^. The absorbance ratio (*A*_420_/*A*_280_) is 0.62. (B) pH dependency of apparent reduction rate constants of the isolated *Mt*DH fragment and the *Mt*CDH holoenzyme in the presence of 30 mm NaCl (open circles) and 30 mm CaCl_2_ (closed circles). For comparison, the apparent reduction rate constants of *Mt*DH in the presence of 30 mm NaCl are shown (grey circles). Traces for FAD reduction were recorded at 449 nm; haem *b* reduction was followed at 563 nm. *Mt*CDH or *Mt*DH (5 μm) were mixed with 3 mm cellobiose, in a stopped-flow spectrometer at a temperature of 30 °C. Indicated concentrations are those after mixing. Values represent means of five independent replicates; error bars are given for all experiments but in many cases are smaller than the data points. (C) Differential scanning calorimetry performed with *Mt*CDH in the presence of 30 mm NaCl or 30 mm CaCl_2_ at pH 5.5 and 7.5. The thermal ramp speed is 1 K min^−1^.

Electron absorption spectra of oxidized *Mt*CDH in the presence of NaCl or CaCl_2_ were recorded at pH 7.5, and showed the typical Soret-band maximum at 421 nm for haem *b* (Fig.3A). Upon reduction, the absorbance of the haem α and β peaks at 533 and 563 nm increased, the Soret band shifted from 421 to 429 nm, and the FAD absorbance at 450 nm decreased. Addition of calcium chloride to a final concentration of 30 mm had no effect on the spectra of the oxidized and reduced enzyme. Measurements at pH 5.5 showed the same result. No indications of Ca^2+^-induced changes in the coordination environment of the haem *b* cofactor were found.

### Inter-domain electron transfer of *Mt*CDH in the presence of CaCl_2_

Based on the spectral characteristics of CDH, stopped-flow spectroscopy was used to monitor reduction of the FAD and haem *b* cofactors of CDH. Rapid mixing of *Mt*CDH (5 μm final concentration) with an excess of cellobiose (3 mm) results in fast FAD reduction, followed by IET and one-electron reduction of the haem *b*. For comparison, the dehydrogenase domain from *M. thermophilum* (*Mt*DH) was also analysed. Figure3B shows apparent pseudo first-order rate constants (*k*_obs_) for both reduction steps between pH 4.5 and 8.5. The FAD reduction changes with pH, and shows a maximum of 38.5 s^−1^ at pH 8.5 in the presence of 30 mm NaCl. In the presence of 30 mm CaCl_2_, *k*_obs_ is only slightly higher and reaches a maximum of 41.6 s^−1^ at pH 7.5. These differences are negligible compared to the changes Ca^2+^ exerts on full-length CDH. The FAD reduction rate in the absence of the dication is almost identical to that in the DH, but in the presence of Ca^2+^, the *k*_obs_ increases 1.3–1.7-fold. The close proximity of the haem *b* appears to exert a pull effect on the FAD, resulting in a faster FAD reduction rate. From docking experiments, a mean edge-to-edge distance of 8 ± 2 Å between FAD and the haem *b* proprionate chain was calculated, which is close enough for fast electron transfer. Modulation of the FAD redox potential by electronic coupling through the bridging Trp295 residue may explain the increased steady-state turnover of DCIP and BQ in the presence of Ca^2+^ (Fig.2E, G and Table2). Both pre-steady-state and steady-state experiments indicate an alkaline pH optimum of approximately pH 8 for the reductive half-reaction at the FAD, which differs greatly from the acidic pH optimum (4.5–5.0) observed for *P. chrysosporium* CDH 9.

The pH-dependent interaction of both CDH domains shows maximal reduction of haem *b* and therefore IET at pH 4.5 (Fig.3B). The pH optimum of *Pc*CDH is slightly lower (pH 3.5 9). In agreement with the steady-state measurements using cyt *c*, an effect of calcium ions was also observed here. Apparent haem reduction rates at pH 4.5 increased from 0.25 to 1.37 s^−1^ upon addition of CaCl_2_. At pH 6.5 and above, the haem reduction became extremely slow. At pH 7.5, only ∼ 5% of haem *b* cofactors was reduced within the observed time span of 30 s, but haem reduction was recovered by addition of Ca^2+^. In the presence of Ca^2+^, *k*_obs_ decreases monotonically from pH 4.5 to 7.5, but the rate is always higher compared to the rates measured in the absence of Ca^2+^. This decrease indicates that, with increasing pH, the cation-bridging effect is overcome by electrostatic repulsion. A comparison with the steady-state cyt *c* pH profile shows that the positive charges of this proteinogenic electron acceptor strongly influence the IET. Turnover maxima of *Mt*CDH for cyt *c* are found at pH 5 in the absence of Ca^2+^ and at pH 8 in the presence of Ca^2+^, whereas the IET measured by stopped-flow in the absence of cyt *c* continuously decreases from its maximum at pH 4.5, in either the presence or absence of Ca^2+^. These results indicate the limits of the use of cyt *c* to measure IET.

### Effect of calcium chloride on the thermostability of *Mt*CDH

**Figure 4:**
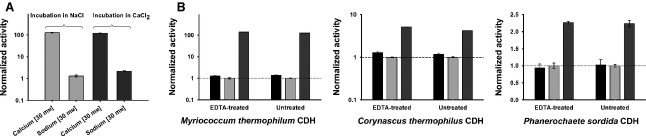
(A) Cyt *c*-dependent activity of *Mt*CDH at pH 7.5 in the presence and absence of 30 mm calcium. The *x* axis indicates the ion concentration in the assay mixture. *Mt*CDH activity was measured before and after incubation in the salt containing solutions at pH 7.5 and room temperature. Error bars indicate the standard deviation of three independent replicates. (B) Comparison of cyt *c* activities of untreated and EDTA-treated CDHs. Black bars, salt-free buffer; light grey bars, addition of 30 mm sodium chloride; dark grey bars, addition of 30 mm calcium chloride. Error bars indicate the standard deviation of three independent measurements. Activities of *Mt*CDH and *Ct*CDH were measured at pH 7.5 (MOPS, 50 mm), whereas that of *Pc*CDH was measured at pH 6.5 (MOPS, 50 mm).

Transition mid-point temperatures (*T*_M_) of *Mt*CDH in the presence of 30 mm NaCl or 30 mm CaCl_2_ were recorded at pH 5.5 and pH 7.5. At pH 5.5, overlapping peaks were observed, with a maximum (*T*_M_) at 68.5 °C (Fig.3C and Table4). At pH 7.5, the presence of CaCl_2_ shifted the thermostability only slightly by 0.7 °C (from 70.7 to 71.4 °C). The observation of a slightly higher *T*_M_ at pH 7.5 shows that the closed conformation at pH 5.5 does not lead to higher thermal stability of the enzyme, which suggests that no strong interactions are involved in forming the closed structure. It also confirms that stabilization of the closed conformation of CDH in the presence of Ca^2+^ is achieved by weak forces rather than strong binding. To further test whether divalent ions exert a permanent interaction with *Mt*CDH, the enzyme was incubated in 30 mm CaCl_2_ at 22 °C for 2 h. Dilution in a buffer containing 30 mm NaCl showed that the activity-enhancing effect disappeared and the activity became similar to that of the negative control incubated in buffer containing 30 mm NaCl (Fig.4A). Incubation of the enzyme in 30 mm NaCl and subsequent measurement of cyt *c* activity in the presence of 30 mm CaCl_2_, resulted in the same enhancement of activity. Based on the results of these experiments, it may be concluded that the nature of the interaction is transient and does not increase the stability of CDH. To exclude potential interferences from residual divalent metal ions in the CDH preparations, CDHs from *Phanerochaete sordida*, *Corynascus thermophilus* and *Myriococcum thermophilum* (0.2 mg·mL^−1^) were incubated with 5 mm EDTA for 1 h at 22 °C. After incubation, enzymes were extensively diafiltered using EDTA-free buffer. The same procedure was applied to cyt *c*. The cyt *c* activities of the EDTA-treated and untreated enzymes were measured, and no difference was observed (Fig.4B), indicating that no interfering ions were present.

**Table 4:** Effect of 30 mm CaCl_2_ and 30 mm NaCl on the stability of *Mt*CDH at pH 5.5 and 7.5 measured by differential scanning calorimetry

	pH 5.5			pH 7.5		
	Buffer	NaCl	CaCl_2_	Buffer	NaCl	CaCl_2_
*T*_M_ (°C)	67.3	68.5	68.5	70.4	70.7	71.4
∆*H* (kJ·moL^−1^)	393	454	473	847	565	634

### Structure analysis and docking of CDH domains

**Figure 5:**
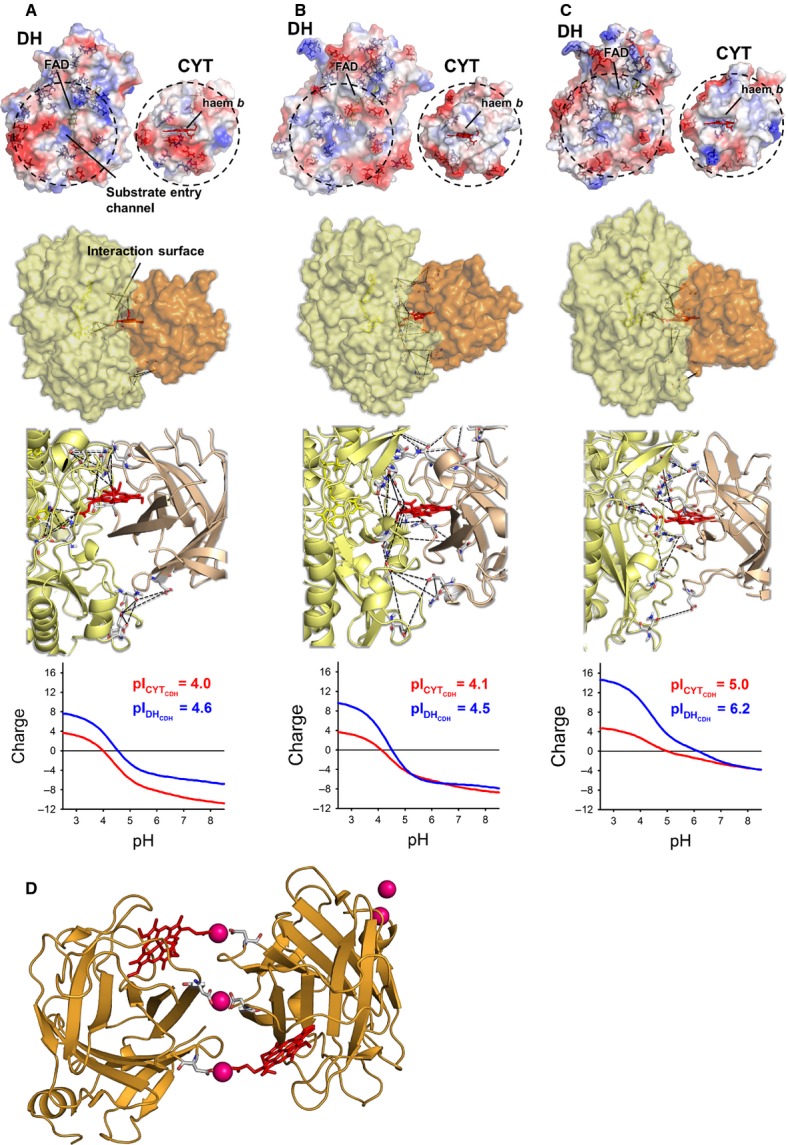
(A–C) Surface charge at pH 7.0, docking models and calculated titration plots of the amino acids located at the interface of *Pc*CDH (A) *Mt*CDH (B) and *Ct*CDH (C). Residues considered for calculation of titration plots are those within circles. *Mt*CDH and *Ct*CDH are homology models based on separately crystallized *Pc*CDH sub-domains (PDB IDs 1NAA and 1D7C). The docking models show the DH in yellow and the CYT in orange. Distances between clusters are used to indicate their position (black lines). (D) Crystal structure of the CYT fragment of *P. chrysosporium*, including the coordination of co-crystallized cadmium atoms (pink).

The characterized *Mt*CDH, together with CDH from *P. chrysosporium* (*Pc*CDH) and *C. thermophilus* (*Ct*CDH), as examples of class I and class II CDHs, were used for modelling and docking studies. To analyse the DH/CYT interface of the CDHs, homology models of *Mt*DH, *Mt*CYT, *Ct*DH and *Ct*CYT were generated, and, together with the crystal structures of *Pc*DH and *Pc*CYT, were subjected to docking. The opposing domain surfaces of each CDH are shown in Fig.5, which highlights charged Asp, Glu, Lys and Arg residues at the domain interface or in its vicinity. *Pc*CDH and *Mt*CDH show the highest number of negatively charged amino acid residues in the interfacial area (five for *Pc*CYT, ten for *Pc*DH, seven for *Mt*CYT and eight for *Mt*DH), whereas *Ct*CDH has fewer charges (five for *Ct*CYT and seven for *Ct*DH). However, the number of charged amino acids alone does little to explain the domain behaviour and IET of the selected CDHs. Therefore, titration plots of the surface-exposed amino acids in the interfacial area were created (Fig.5). The calculated isoelectric points are similar for *Pc*CDH and *Mt*CDH (∼ 4.0 for the CYT, ∼ 4.5 for the DH), consistent with experimental data that show the same upper pH limit of IET for both enzymes (6). The titration plots also correctly predict a higher pH for electrostatic repulsion of the domains for *Ct*CDH (pI of ∼ 5.0 for the CYT and ∼ 6.2 for the DH), which does indeed show a higher pH for its IET optimum. However, the two-dimensional view does not give information on the divalent cation-bridging effect. Therefore, the interface of the docking models was investigated. The obtained docking positions do not differ by much, but all models were evaluated, and the best was selected on the basis of (i) a close haem *b* to FAD distance, and (ii) a suitable length and orientation of the linker peptide. Interestingly, the distance between the haem *b* propionate group reaching into the substrate channel and the FAD isoalloxazine ring (edge-to-edge distance) was shortest for *Ct*CDH (5.6 ± 0.6 Å) compared to *Mt*CDH and *Pc*CDH (8 ± 2 and 8.6 ± 2 Å, respectively). A closer distance between the cofactors in the closed confirmation may additionally support the occurrence of IET at an increased pH under which the domains begin to separate. The mean calculated interfacial area between the DH and the CYT for the three CDHs differs by a maximum of 16% (*Mt*CDH 2270 ± 280 Å^2^, *Ct*CDH 2090 ± 70 Å^2^, *Pc*CDH 1910 ± 250 Å^2^). Docked structures with highlighted cofactors and distances below 13 Å between opposing Asp, Glu, Asn and Gln residues are shown in Fig.5. Such clusters, which inadequately mimic the structurally well-defined Ca-binding sites in calmodulin, for example, allow weak electrostatic interactions between Ca^2+^ and negatively charged amino acid side chains. It is obvious that, in *Mt*CDH, these clusters contain many residues, but in *Pc*CDH and *Ct*CDH, they contain only a few. Four clusters were found in *Mt*CDH and *Pc*CDH, and three in *Ct*CDH (Table S2). For all enzymes, at least one cluster involves one or both haem propionate groups. The much higher number of potential dication ligands in *Mt*CDH provides an explanation for the high increase of IET at alkaline pH. The presence of more sites for Ca^2+^ binding more efficiently counteracts electrostatic repulsion of the domains. Interestingly, for a number of proteins crystallized in the presence of cadmium ions, it has been observed that Cd^2+^ is positioned at the interface between two neighboring protein molecules and coordinated by two carboxyl groups of glutamic or aspartic acid side chains each belonging to one of the two molecules 23–25. This coordination may be supplemented by interactions with carbonyl groups of the polypeptide backbone. Such complexation was also observed in the structure of *P. chrysosporium* CYT (PDB ID 1D7C 13). Either one or both of the Cd^2^ ^+^ -complexing sites known for dimers of *P. chrysosporium* CYT (Fig.5D) were found in the predictions for *Pc*CDH, *Mt*CDH and *Ct*CDH.

## Conclusions

Steady-state and pre-steady-state measurements in combination with homology models show that the pH dependence of IET and the reduction of cyt *c* may be explained by the presence of negatively charged amino acids at the interface of the DH and CYT, preventing formation of a closed conformation of the DH and the CYT. Calculated titration plots of these interfaces demonstrate that the CYT has a lower pI than the DH; the same trend was also observed for the isoelectric points of the domains 10. Below, or a little above these pIs, the domains do not repulse each other, and thus allow electron transfer between the domains.

In the present work, we also unequivocally demonstrate that dications enhance the IET reaction in cellobiose dehydrogenase, as first suggested by Schulz *et al*. 14. Catalytic constants for cyt *c* at pH 7.5 measured in the presence of calcium ions indicate that the presence of divalent cations supports a closed conformation of both domains at neutral and alkaline pH. The reduction state of the cofactors during catalysis, monitored by stopped-flow spectroscopy, reveals that Ca^2+^ affects the IET most strongly. Less prominent, but clearly observable, is the coupling of both cofactors in close proximity of the domains, which increases the reduction rate of FAD. This effect occurs either at low pH or in the presence of Ca^2+^. Currently, we have no supported explanation for this phenomenon, which was observed in steady-state and pre-steady-state kinetic measurements, but the observed pull effect on the FAD may originate from a modified redox potential of the FAD via an intermediary Trp295 side chain.

It has been reported 18–22 that divalent cations potentially form a bridge between oppositely charged carboxylate groups on polyelectrolytes, DNA or hyaluronic acid. Based on our data, this effect also influences domain interaction in CDH, and presumably also applies to other proteins, given that a number of negatively charged amino acid residues are present in close proximity at domain interfaces. In addition to providing an explanation for the observed pH-dependent IET of CDH and the Ca^2+^ ion effect, these findings may also have practical relevance, e.g. for increasing the current output of CDH-based biosensors or biofuel cells. Also, in accordance with the results of a previous study 26, a CDH-based bioelectrochemical switch may be accomplished at neutral/alkaline pH using divalent cations to switch the IET and hence direct electron transfer on and off. The high millimolar dication concentrations required for such an effect are rarely encountered in nature, but may provide ways to stabilize or regulate enzymes in biocatalytic and biomedical applications.

## Experimental procedures

### Chemicals and enzymes

Chemicals were purchased from commercial suppliers at the highest purity available. The CDHs used in this study are from the fungi *Corynascus thermophilus* CBS 174.70 (*Ct*CDH, 11), *Chaetomium atrobrunneum* CBS 238.71 11, *Hypoxylon haematostroma* CBS 255.63 11, *Dichomera saubinetii* CBS 990.7 11, *Trametes villosa* CBS 334.49 27, *Phanerochaete chrysosporium* K3 (*Pc*CDH 28), *Phanerochaete sordida* MB66 (R. Ludwig, unpublished data), *Sclerotium rolfsii* CBS 191.62 29 and *Ceriporiopsis subvermispora* FP-90031 30. CDHs were isolated and purified as described 11 previously. CDH IIA and IIB from *Neurospora crassa* CBS 232.56 6 and CDH IIA from *Myriococcum thermophilum* CBS 208.89 (*Mt*CDH 31) were recombinantly produced in *Pichia pastoris* X-33 cells, and purified as described 6 previously. The recombinant dehydrogenase domain of *Mt*CDH (*Mt*DH) was produced in *P. pastoris* X-33 and purified as described previously 6.

### Steady-state kinetics

CDH activities were assayed spectrophotometrically based on the cellobiose-dependent reduction of the two-electron acceptors DCIP (0.3 mm, ε_520_ = 6.9 mm^−1^·cm^−1^) and BQ (1 mm, ε_290_ = 2.24 mm^−1^·cm^−1^) and the one-electron acceptors cyt *c* (0.02 mm, ε_550_ = 19.6 mm^−1^·cm^−1^), potassium ferricyanide (1 mm, ε_420_ = 0.98 mm^−1^·cm^−1^) and ferrocenium hexafluorophosphate (0.1 mm, ε_300_ = 4.3 mm^−1^·cm^−1^). All assays contained 3 mm cellobiose, and were performed in 50 mm sodium formiate buffer (pH 3–4), 50 mm sodium acetate buffer (pH 4–6) or 50 mm MOPS buffer (pH 6–8.5). Reactions were started by mixing 20 μL of enzyme solution (0.05–0.2 U·mL^−1^) with 980 μL of a pre-warmed reaction mix containing the electron acceptors and cellobiose. Reduction of electron acceptors was followed for 180 s at 30 °C in a Lambda 35 spectrophotometer (Perkin Elmer, Waltham, MA, USA) with a thermo-controlled eight-cell changer. Enzyme activity is defined as the amount of enzyme that reduces 1 μmol of the respective electron acceptor per minute under the specified conditions. Catalytic constants were calculated from initial rates by using non-linear least-squares regression to fit the observed data to the Michaelis–Menten equation using sigma plot 11 (Systat Software, San Jose, CA, USA). The influence of various anions and cations on CDH activity was measured in 96-well plates in an EnSpire multimode plate reader (Perkin Elmer). For measurements below 340 nm, UV-transparent 96-well-plates or UV-transparent cuvettes were used. Protein concentrations were determined by the dye-binding method 32 using a pre-fabricated assay (Bio-Rad, Hercules, CA, USA) with BSA as the calibration standard. Concentrations of *Mt*CDH used for stopped-flow spectroscopy were determined based on the enzyme’s absorption coefficient at 421 nm (ε_420_ = 99.4 mm^−1^·cm^−1^).

### Pre-steady-state kinetics

Fast kinetic studies were performed on a SX-18MV spectrophotometer (Applied Photophysics, Leatherhead, UK). The cellobiose-dependent reduction of CDH was monitored in single mixing mode using a SX/PDA photodiode array detector or a SX/PMT photomultiplier tube. The reduction of FAD was followed at 449 nm and the reduction of haem *b* was followed at 563 nm. Concentrations of CDH and cellobiose after mixing were 5 μm and 3 mm, respectively. All measurements were performed at 30 °C with at least five repeats for each investigated condition. Observed rate constants (*k*_obs_) were calculated by fitting the absorbance changes to a double exponential curve using pro-data software (Applied Photophysics).

### Differential scanning calorimetry

The transition mid-point temperature (*T*_M_) of *Mt*CDH in the presence of CaCl_2_ and NaCl was determined using a MicroCal VP-DSC calorimeter equipped with an autosampler (MicroCal, Northampton, MA, USA). *Mt*CDH was adjusted to a concentration of 1 mg·mL^−1^ based on its molar absorption at 421 nm. Thermograms were obtained at pH 4.5 (100 mm sodium acetate buffer) and pH 7.5 (100 mm MOPS buffer) between 30 °C and 100 °C at a scan speed of 1 °C min^−1^. Heat-inactivated enzymes were re-scanned as a control, and their values were subtracted from the experimental thermograms. Data were evaluated using origin 7.5 software (Origin Lab Corporation, Northampton, MA, USA).

### Comparative modelling and docking

Structure-guided homology models of the individual DH and CYT of *Mt*CDH (GenBank accession number ABS45567.2) and *Ct*CDH (GenBank accession number ADT70772.1) were generated using the SWISS-MODEL server (http://swissmodel.expasy.org/) 33 with the individually crystallized DH of *P. chrysosporium* CDH (PDB ID 1KDG 12) and the CYT of *P. chrysosporium* CDH (PDB ID 1D7C 13) as templates. The HADDOCK webserver (http://haddock.science.uu.nl/) was used for protein–protein docking of the individual CDH domains 34. To roughly pre-define the docking position, residues located within 4 Å of the haem *b* CYT (Pro72, Tyr98, M100, Gln173, Gln174, His175 and Met179) and residues in proximity to the substrate channel in the DH (Met80, Ala81 and Val91) were selected as initial docking sites. In total, 12 models from three clusters per enzyme were evaluated. Models with a haem *b* to FAD distance of more than 10 Å were not considered for further analysis. Structures were visualized using the PyMOL molecular graphics system, version 1.4 (Schrödinger, New York, NY, USA).

## Abbreviations

BQ: 1,4-benzoquinone
CDH: cellobiose dehydrogenase
CYT: cytochrome domain
DCIP: 2,6-dichloroindophenol
DH: dehydrogenase domain
IET: inter-domain electron transfer

## Supporting Information

**Table S1.** Influence of anions and cations (30 mm) on *Mt*CDH activity measured using cyt *c*, DCIP and 1,4-benzoquinone. **Table S2.** Distances between Asp, Glu, Gln and Asn residues in the docking models.
