# Supplementary material for: Inter-domain electron transfer in cellobiose dehydrogenase: modulation by pH and divalent cations
Source: FEBS J. 2015 May 16;282(16):3136–48. doi: 10.1111/febs.13310 (PMC4676925; doi:10.1111/febs.13310)
Supplement: Supplementary file 1 [file febs0282-3136-sd1.zip › febs13310-sup-0001-TableS1-S2.pdf]

the FEBS Journal
www.febsjournal.org

Inter-domain electron transfer in cellobiose dehydrogenase: modulation by pH and divalent cations

Daniel Kracher, Kawah Zahma, Christopher Schulz, Christoph Sygmund, Lo Gorton and Roland Ludwig

DOI: 10.1111/febs.13310

Table S1. Influence of different anions and cations (30 mM) on *Mt*CDH activity measured with cyt *c*, DCIP and 1,4-benzoquinone .

Salt	Cyt *c*		DCIP		1,4-Benzoquinone	
	pH 5.5	pH 7.5	pH 5.5	pH 7.5	pH 5.5	pH 7.5
**Blank**	100.0 ± 5.2	100.0 ± 0.9	100.0 ± 3.7	100.0 ± 3.6	100.0 ± 1.0	100.0 ± 3.3
**LiCl**	96.5 ± 5.0	126.0 ± 1.0	96.5 ± 11.3	98.1 ± 5.1	113.5 ± 0.4	103.3 ± 9.6
**NaCl**	92.3 ± 7.0	86.9 ± 0.9	93.9 ± 9.4	100.9 ± 9.1	92.5 ± 1.2	105.3 ± 1.4
**KCl**	96.1 ± 4.7	93.2 ± 6.9	104.2 ± 3.5	100.0 ± 13.0	104.6 ± 7.4	104.0 ± 3.0
**RbCl**	93.8 ± 2.1	97.3 ± 1.3	93.9 ± 14.1	92.9 ± 2.5	101.0 ± 1.0	107.7 ± 13.0
**NaF**	96.4 ± 1.6	65.0 ± 0.4	103.4 ± 8.1	93.6 ± 6.2	103.7 ± 1.9	105.9 ± 7.2
**KBr**	87.2 ± 2.1	114.9 ± 4.7	103.2 ± 4.4	96.2 ± 3.4	110.6 ± 5.4	109.1 ± 6.5
**$NaCH_3COO$**	111.1 ± 1.1	90.5 ± 1.0	99.4 ± 4.0	88.3 ± 13.5	110.1 ± 5.4	106.8 ± 5.6
**KSCN**	92.9 ± 0.9	69.9 ± 2.0	96.1 ± 10.1	77.0 ± 2.4	97.0 ± 3.6	108.3 ± 3.5
**Na_2SO_4**	104.6 ± 7.8	89.6 ± 2.1	94.0 ± 16.8	82.9 ± 2.9	111.2 ± 1.8	100.9 ± 4.4
**K_2PO_4**	102.8 ± 7.9	84.7 ± 1.0	91.4 ± 23.3	79.3 ± 4.2	100.1 ± 2.2	110.3 ± 2.1
**Oxalate**	128.5 ± 4.4	97.2 ± 1.3	102.6 ± 5.4	111.8 ± 6.7	108.0 ± 3.9	107.4 ± 13.3
**Succinate**	116.5 ± 1.9	102.7 ± 2.7	97.2 ± 9.6	89.4 ± 2.8	119.2 ± 0.2	107.7 ± 7.6
**Citrate**	139.2 ± 5.8	100.0 ± 1.6	97.5 ± 7.7	87.6 ± 0.6	105.9 ± 2.5	109.2 ± 3.2
**Na_3BO_3**	97.0 ± 3.3	110.3 ± 6.0	99.1 ± 8.5	102.8 ± 3.1	105.3 ± 1.0	106.2 ± 12.2

Standard deviations are calculated from three repeats.
Assays supplemented with acids or bases were titrated to 5.5 or 7.5 by means of NaOH or phosphoric acid.

Table S2. Distances of Asp, Glu, Gln and Asn in clusters found in the docking models.

***Mt*CDH**	**DH**	**CYT**	**Distance (Å)**
**Cluster 1**	D307	P2	6.9
	Q308	P2	5.7
	D307	P1	11.7
	Q308	P1	8.0
	N696	P2	9.5
	N696	P2	5.4
**Cluster 2**	D305	N74	10.8
	E304	N74	10.2
	E304	E104	11.1
	E304	D101	7.4
**Cluster 3**	Q293	D159	12.3
	D297	Q174	9.5
	Q293	D159	12.3
	D297	D159	12.8
	Q293	Q173	7.0
	D297	Q174	9.5
**Cluster 4**	E603	D24	8.5
	E603	Q27	12.9
	N678	D24	11.7

***Pc*CDH**	**DH**	**CYT**	**Distance (Å)**
**Cluster 1**	N687	P1	5.2
	N685	P1	4.8
	D515	P1	8.9
**Cluster 2**	D283	E162	8.9
	N285	E162	10.7
**Cluster 3**	D622	P2	5.7
	D292	P2	10.9
	D292	N66	8.5
	D622	N66	9.3
**Cluster 4**	Q670	D24	12.1
	N592	D24	8.0

***Ct*CDH**	**DH**	**CYT**	**Distance (Å)**
**Cluster 1**	D523	P1	9
	N694	P1	4.1
**Cluster 2**	Q303	P2	5.0
	D302	P2	7.8
	Q303	Q66	3.6
	D302	Q69	5.9
	D300	Q69	10.6
	D302	Q66	4.8
**Cluster 3**	D598	Q175	8.5
	N690	Q175	9.1
